# Temporal Variability and Ecological Interactions of Parasitic Marine Syndiniales in Coastal Protist Communities

**DOI:** 10.1128/mSphere.00209-20

**Published:** 2020-05-27

**Authors:** Sean R. Anderson, Elizabeth L. Harvey

**Affiliations:** a Skidaway Institute of Oceanography, University of Georgia, Savannah, Georgia, USA; b University of New Hampshire, Durham, New Hampshire, USA; Clemson University

**Keywords:** Syndiniales, microbial interactions, network analysis, parasitism, protists

## Abstract

Syndiniales are a ubiquitous group of protist parasites that infect and kill a wide range of hosts, including harmful bloom-forming dinoflagellates. Despite the importance of parasitism as an agent of plankton mortality, parasite-host dynamics remain poorly understood, especially over time, hindering the inclusion of parasitism in food web and ecosystem models. For a full year in the Skidaway River Estuary (Georgia), we employed weekly 18S rRNA sampling and co-occurrence network analysis to characterize temporal parasite-host infection dynamics of Syndiniales. Over the year, Syndiniales exhibited strong temporal variability, with higher relative abundance from June to October (7 to 28%) than other months in the year (0.01% to 6%). Nonmetric dimensional scaling of Syndiniales composition revealed tight clustering in June to October that coincided with elevated temperatures (23 to 31°C), though in general, abiotic factors poorly explained composition (canonical correspondence analysis [CCA] and partial least-squares [PLS]) and were less important in the network than biotic relationships. Syndiniales amplicon sequence variants (ASVs) were well represented in the co-occurrence network (20% of edges) and had significant positive associations (Spearman *r *> 0.7), inferred to be putative parasite-host relationships, with known dinoflagellate hosts (e.g., *Akashiwo* and *Gymnodinium*) and other protist groups (e.g., ciliates, radiolarians, and diatoms). Positive associations rarely involved a single Syndiniales and dinoflagellate species, implying flexible parasite-host infection dynamics. These findings provide insight into the temporal dynamics of Syndiniales over a full year and reinforce the importance of single-celled parasites in driving plankton population dynamics. Further empirical work is needed to confirm network interactions and to incorporate parasitism within the context of ecosystem models.

**IMPORTANCE** Protist parasites in the marine alveolate group, Syndiniales, have been observed within infected plankton host cells for decades, and recently, global-scale efforts (Tara Ocean exploration) have confirmed their importance within microbial communities. Yet, protist parasites remain enigmatic, particularly with respect to their temporal dynamics and parasite-host interactions. We employed weekly 18S amplicon surveys over a full year in a coastal estuary, revealing strong temporal shifts in Syndiniales parasites, with highest relative abundance during warmer summer to fall months. Though influenced by temperature, Syndiniales population dynamics were also driven by a high frequency of biological interactions with other protist groups, as determined through co-occurrence network analysis. Parasitic interactions implied by the network highlighted a range of confirmed (dinoflagellates) and putative (diatoms) interactions and suggests parasites may be less selective in their preferred hosts. Understanding parasite-host dynamics over space and time will improve our ability to include parasitism as a loss term in microbial food web models.

## Abstract (precis)

Protist parasites in the marine alveolate group, Syndiniales, have been observed within infected plankton host cells for decades, and recently, global-scale efforts (Tara Ocean exploration) have confirmed their importance within microbial communities. Yet, protist parasites remain enigmatic, particularly with respect to their temporal dynamics and parasite-host interactions. We employed weekly 18S amplicon surveys over a full year in a coastal estuary, revealing strong temporal shifts in Syndiniales parasites, with highest relative abundance during warmer summer to fall months. Though influenced by temperature, Syndiniales population dynamics were also driven by a high frequency of biological interactions with other protist groups, as determined through co-occurrence network analysis. Parasitic interactions implied by the network highlighted a range of confirmed (dinoflagellates) and putative (diatoms) interactions and suggests parasites may be less selective in their preferred hosts. Understanding parasite-host dynamics over space and time will improve our ability to include parasitism as a loss term in microbial food web models.

## INTRODUCTION

Marine microbial eukaryotes (i.e., protists) occupy diverse and ecologically important roles within marine food webs, as they represent both primary producers and consumers of organic carbon (1, 2). Protist abundance, diversity, and composition are driven by processes that either stimulate growth or promote mortality. In terms of mortality, the magnitudes of loss rates are often determined by interactions that occur between individual species (e.g., predator-prey or parasite-host) and with the surrounding environment (3, 4). Identifying and quantifying these interactions are essential, as the type of interaction can dictate how carbon and nutrients are cycled through the food web (2, 3). While grazing by heterotrophic protists and infection by viruses have been identified as major sources of plankton mortality across global oceans (5, 6), parasitism is also widespread within protist communities, may at times exceed grazing mortality, and has been described an as important interaction among protists (7–9).

Parasites of phytoplankton and microzooplankton (including mixotrophs) represent important agents of mortality in marine systems, influencing plankton bloom dynamics, species succession, and host biodiversity and evolution (8, 10). One major group of unicellular parasites, Syndiniales (marine alveolates), are ubiquitous, often dominating the relative abundance in global protist communities (11–14) and accounting for the bulk of biotic interactions inferred by sequence-based correlation networks (15–17). The ubiquity of these protists may be related to their life history, which typically involves a short-lived intracellular trophont stage (2 to 3 days), followed by the release of hundreds of free-living dinospores (<10 μm) that can survive outside the host for 1 to 2 days (18). Parasite dinospores readily infect and kill a wide range of hosts, including other protists (e.g., dinoflagellates, ciliates, and radiolarians) and metazoans (11). Moreover, the impact of parasitic infection on biogeochemical cycles differs from other forms of mortality, such as grazing, as parasitism reroutes a portion (up to 50%) of host biomass away from the traditional food web (akin to viral lysis), supplying carbon and nutrients to the microbial loop (19–21). Therefore, a deeper understanding of Syndiniales infection dynamics is warranted, especially given the impact their life history (e.g., abundant progeny and short generation times) can have on plankton populations and related carbon flow within microbial food webs.

Though recent work has confirmed the global distributions of Syndiniales (e.g., Tara Oceans exploration [12]), the ability to incorporate parasitism within food web and ecosystem models has remained challenging, hindered by a lack of understanding of *in situ* protist parasite dynamics, including temporal shifts in parasite populations and parasite-host interactions. For instance, only a few studies have considered temporal variability (on monthly scales) in Syndiniales at a single location (16, 22), and often, sampling has favored summer months, where elevated host concentrations improve the chances of detecting host infection (23). Indeed, host density is thought to be the main driver of parasite abundance and infection rates, with increased encounters and infection of hosts occurring under plankton bloom conditions (20, 24). Other factors may influence Syndiniales population dynamics such as temperature (17, 19), nutrients (25), water column depth (24), and degree of physical mixing (26), though such factors have not been examined over broad time scales. Nevertheless, such limited temporal resolution has made it difficult to identify reliable drivers of Syndiniales populations over a range of environmental and biological conditions.

Syndiniales infection dynamics, including host specificity and preference, also remain ambiguous, likely related to the enormous diversity within Syndiniales (>50 clades across five main groups) and the fact that most parasite sequences have yet to be taxonomically classified or brought into culture (11, 15, 27). Syndiniales are often associated with coastal plankton blooms, including frequent observations of Syndiniales within the genus *Amoebophyra*, infecting harmful bloom-forming dinoflagellates such as *Akashiwo* or *Alexandrium* (23, 24, 28–30). Documented parasitic interactions exhibit a range in specificity, as *Amoebophyra* spp. have been shown to selectively parasitize a single dinoflagellate species in the field (31), though in culture, several *Amoebophyra* sp. strains are capable of infecting multiple hosts (18, 32, 33). A generalist or opportunistic infection strategy may be common, as environmental sequences within Syndiniales are often correlated with multiple hosts (across different classes) via network analysis and are widely distributed in marine ecosystems (11, 15, 16). Establishing parasite-host dynamics in a natural setting will provide key insights into the specificity of parasitic interactions and will benefit from consistent and high-throughput sampling of *in situ* protist communities.

Coastal estuaries are an ideal location to examine parasite-host dynamics over time, as temporal changes (hourly or monthly) in abiotic factors often lead to ephemeral plankton blooms (including harmful microalgae) that are susceptible to parasitic infection (28). Here, we investigated temporal shifts in coastal Syndiniales communities, measuring Syndiniales relative abundance, diversity, and community composition through V4 18S rRNA gene tag sequencing. Our survey consisted of 33 weekly or biweekly surface water samples collected over a full year within the Skidaway River Estuary (Georgia, USA), a shallow and productive subtropical estuary with wide environmental gradients (34). To visualize significant interactions between Syndiniales and putative protist hosts, we applied co-occurrence analysis, which has become an important tool for inferring meaningful associations (e.g., parasitism, grazing, or competition) between taxonomic sequences (35, 36). Our specific objectives through this work were to (i) explore abiotic and biotic drivers of Syndiniales over realistic and more resolved time scales and (ii) determine significant associations between Syndiniales and putative hosts that may imply parasitism and assess the specificity of such associations. Given that infection by Syndiniales parasites can elicit strong top-down pressure on plankton populations, alter microbial diversity and coevolution, and influence carbon cycling (8), gathering baseline knowledge of protist parasite dynamics will be critical to inform parasitic mortality in coastal ecosystem and biogeochemical models.

## RESULTS

### Temporal variability in Syndiniales.

**FIG 1:**
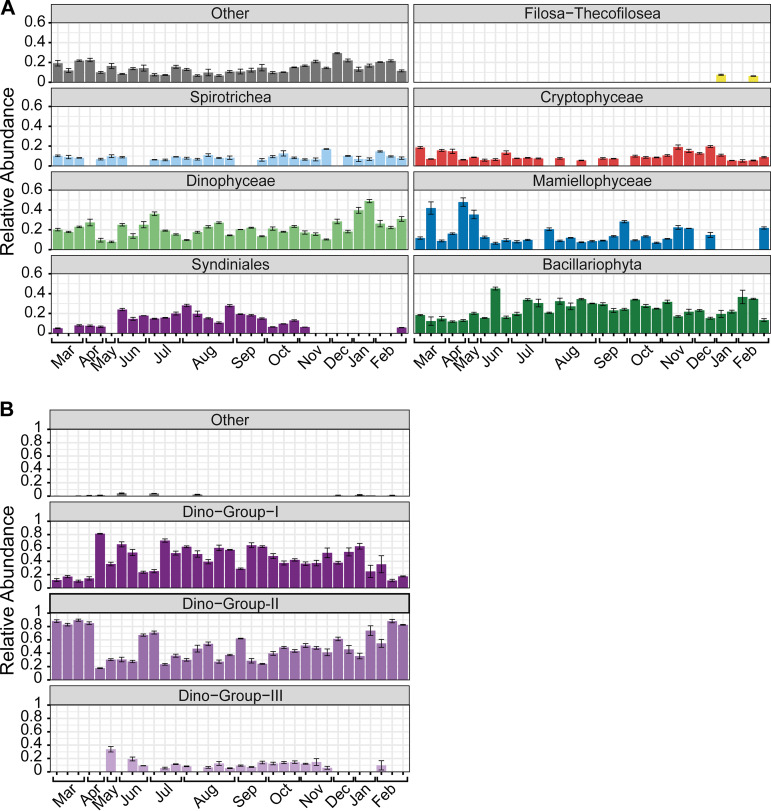
Relative abundance bar plots of major taxa in the estuary over the year, according to PR2 annotation at the class level (A) and order level (B) within the Syndiniales group. Bar plots are faceted and not stacked to visualize temporal trends for each protist group. Error bars represent the standard deviations from replicate sample means. Taxa included in the “other” category represent <5% of the total protist or Syndiniales community on each respective day. Syndiniales were dominated by three groups (Dino-Groups I, II, and, III), with only a few sequences being unclassified at the order level. Samples from the same month are indicated within brackets on the *x* axis for all temporal figures (see Table S1 in the supplemental material for exact dates).

Over the year, the average relative abundance at the class level was dominated by Bacillariophyta (24%) and Dinophyceae (21%), while other groups such as Mamiellophyceae (14%), Syndiniales (10%), Cryptophyceae (9%), Spirotrichea (8%) and Filosa-Thecofilosea (2%) were less abundant (Fig. 1A). However, of all the major protist groups (i.e., relative abundance of >5% on any day), only Syndiniales exhibited strong temporal variation, with highest relative abundance from June to October (7% to 28%) compared to that in other months in the year (<0.01% to 6%) (Fig. 1A). Syndiniales (total 658 amplicon sequence variants [ASVs]) were composed of three main groups at the order level (here, termed Dino-Groups I, II, and III). The observed June to October peak in Syndiniales abundance was mainly attributed to ASVs in Dino-Groups I and II (>60% of Syndiniales abundance), though ASVs from Group III exhibited temporal variability and emerged at this time (0.02% to 19%) (Fig. 1B).

10.1128/mSphere.00209-20.1 FIG S1 Rarefaction curves generated for the total 18S community. Curves are faceted by month and include all replicated samples within a given month. Download FIG S1, TIF file, 0.9 MB. Copyright © 2020 Anderson and Harvey. 2020 Anderson and Harvey This content is distributed under the terms of the Creative Commons Attribution 4.0 International license.

10.1128/mSphere.00209-20.6 TABLE S1 Raw data for environmental variables measured on each sampling day over the year, as well as variable influence of the projection (VIP) values from partial least squares (PLS) regressions with each protist group. POC/PON, particulate organic carbon or nitrogen. PLS models were run using average relative abundances. Most important VIP values (VIP > 1) are bolded. Download Table S1, XLSX file, 0.1 MB. Copyright © 2020 Anderson and Harvey. 2020 Anderson and Harvey This content is distributed under the terms of the Creative Commons Attribution 4.0 International license.

Canonical correspondence analysis (CCA) and partial least-squares (PLS) regression were performed to assess relationships between environmental variables and protist composition or relative abundance, the latter accounting for observed collinearity in variables. CCA revealed temporal variability in protist communities over the year, with environmental factors explaining 19% of the variance (sum of CCA1 and CCA2 axes) for both total 18S and Syndiniales communities (see Fig. S2 in the supplemental material). For both CCA plots, communities in June to October corresponded most closely to temperature and silicate (Fig. S2). Of the variables included, PLS models identified temperature (variable influence on the projection [VIP] = 1.43) and silicate (VIP = 1.57) to be the most important in explaining relative abundance of Syndiniales at the class level (see Table S1), confirming CCA analyses. Temperature was found to be an important factor (VIP > 1), explaining shifts in relative abundances from most protist groups, except for Bacillariophyta abundance, which was most explained by dissolved nutrients (Table S1).

10.1128/mSphere.00209-20.2 FIG S2 Canonical correspondence analysis (CCA) based on total 18S composition (A) and Syndiniales composition (B). Environmental factors are represented by arrows. Temp, temperature (°C); Sal, salinity; SiO_4_, silicate (μM); NO_3_, nitrate (μM); NH_4_, ammonium (μM); PO_4_, phosphate (μM); Chl, chlorophyll (μg liter^−1^); DO, dissolved oxygen (mg liter^−1^); POC/PON, particulate organic carbon or nitrogen (μg C or N liter^−1^); Solar, solar radiation (MJ m^−2^). Replicates are shown for each sampling day and colored by month. Download FIG S2, TIF file, 1.7 MB. Copyright © 2020 Anderson and Harvey. 2020 Anderson and Harvey This content is distributed under the terms of the Creative Commons Attribution 4.0 International license.

**FIG 2:**
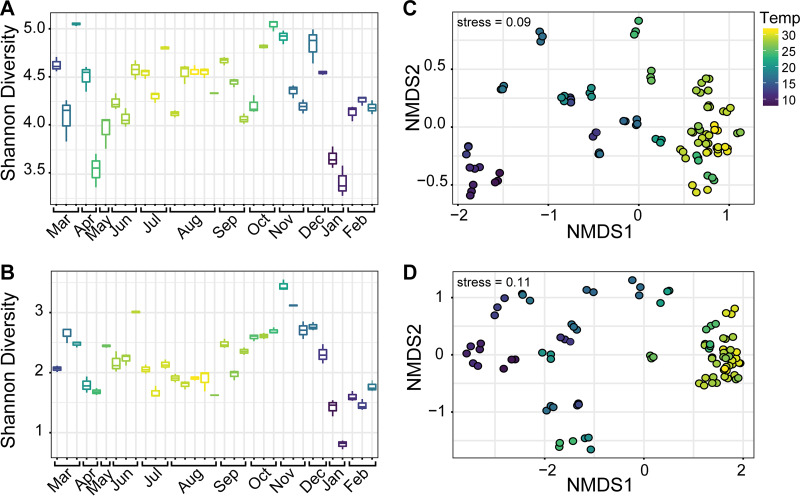
Temporal shifts in Shannon alpha diversity and ordination by nonmetric dimensional scaling (NMDS) of the total protist community (A and C) and only Syndiniales (B and D). Alpha diversity values represent mean and standard deviations from replicate samples, while points in the NMDS represent Bray-Curtis distances of communities (based on relative abundance) for each replicate per sample. Sampling days are colored similarly for both diversity metrics according to the observed temperature gradient in the estuary. Stress values for the NMDS are shown.

Given the wide temperature gradient in the estuary (6 to 31°C) and its importance as an environmental driver of composition (37), we used temperature in favor of arbitrarily binning samples (e.g., by season) to more accurately visualize temporal shifts in diversity (Fig. 2). Shannon diversity values for both total 18S and Syndiniales-only sequences were stable over the year, except for a few samples in April and January, where alpha diversity decreased (Fig. 2A and B). The diversity among Syndiniales was driven largely by Dino-Group II, which was represented by 32 clades, while Dino-Group I included only four clades (see Table S2). Nonmetric dimensional scaling (NMDS) of the total 18S community revealed considerable clustering of samples collected in temperatures ranging from 23 to 31°C (June to October), whereas samples from colder temperatures were more scattered (Fig. 2C). Communities within Syndiniales exhibited even tighter temperature-based clustering in the warmer months from June to October (Fig. 2D).

10.1128/mSphere.00209-20.7 TABLE S2 Taxonomic assignments and corresponding sequences for protist ASVs following filtering steps (e.g., singletons and metazoans removed). Relative abundances of each ASV are shown per replicate and sampling day over the year. Download Table S2, XLSX file, 4.1 MB. Copyright © 2020 Anderson and Harvey. 2020 Anderson and Harvey This content is distributed under the terms of the Creative Commons Attribution 4.0 International license.

### Protist co-occurrence networks.

**FIG 3:**
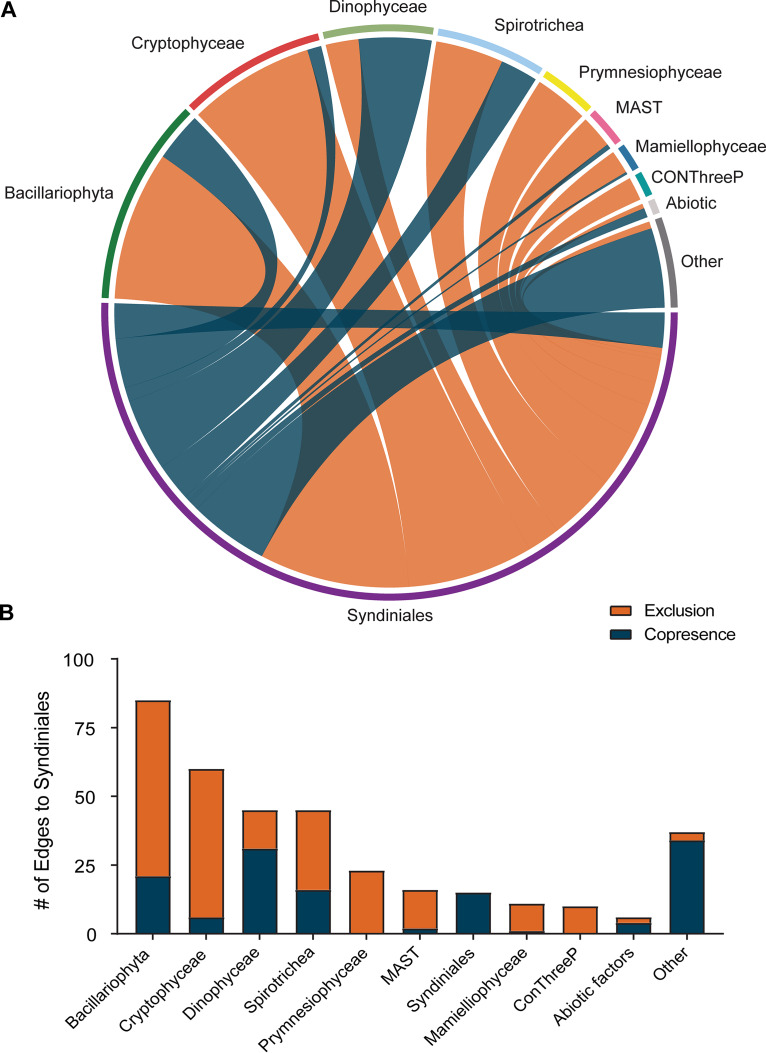
(A) Filtered co-occurrence network of positive (copresence; blue) and negative (exclusion; orange) edges between Syndiniales ASVs and ASVs from other protist groups. ASVs included in the network were found in >50% of samples. Class-level groups or abiotic factors associated with Syndiniales (labeled nodes) were represented by 2 to 21 ASVs per group. The following classes were represented by <2 ASVs per class and included in the “other” group: Bioecea, Chlorophyceae, Filosa-Thecofilosea, Katablepharidaceae, Nephroselmidophyceae, Pedinophyceae, Porphyridiophyceae, Pyramimonadales, and Trebouxiophyceae. Thicker lines represent more interactions found between Syndiniales and other protist groups. Edges were computed between ASVs based on a suite of correlation and similarity metrics and included if statistically significant (merged *q* value < 0.05). (B) Total numbers of positive and negative edges connected between Syndiniales and other major groups.

We constructed a co-occurrence network which described significant relationships between the most abundant 152 protist ASVs (represented as nodes) observed in the estuary over the year. Environmental data were also included to assess relationships between specific ASVs and abiotic factors (e.g., 7 additional nodes). The initial network consisted of 1,793 relationships (or edges) connected between 150 nodes, with the majority (64%) of edges being negative (exclusion) associations (see Fig. S3; Table S3). Filtering of the network to include only the edges related to Syndiniales (12 ASVs) accounted for 20% of all edges found in the overall protist network (Fig. 3A; see also Table S4). Significant edges (*q* values < 0.05) were observed between Syndiniales and a range of other class level groups, including Bacillariophyta (21 ASVs), Dinophyceae (11 ASVs), Cryptophyceae (9 ASVs), and Spirotrichea (8 ASVs) (Fig. 3A). Syndiniales ASVs were most often associated with Bacillariophyta (85 total edges) and Cryptophyceae (60 total edges), with edges typically representing negative associations between ASVs (Fig. 3B).

10.1128/mSphere.00209-20.3 FIG S3 Positive (copresence; A) and negative (exclusion; B) interactions inferred by the total 18S protist network. ASVs included in the network were found in >50% of samples. Nodes were grouped on the outside the circle by class (or abiotic factors) and poorly represented taxa (<2 ASVs per class) were combined into “other” category and included the following: Acantharea, Bioecea, Cercozoa_X, Chlorodendrophyceae, Chlorophyceae, Filosa-Thecofilosea, Filosa-Imbricatea, Katablepharidaceae, Nephroselmidophyceae, Pedinophyceae, Planomonadida, Porphyridiophyceae, Pyramimonadales, Trebouxiophyceae, and Ulvophyceae. Thickness of the line indicates number of positive or negative interactions between connected groups. Download FIG S3, TIF file, 2.7 MB. Copyright © 2020 Anderson and Harvey. 2020 Anderson and Harvey This content is distributed under the terms of the Creative Commons Attribution 4.0 International license.

10.1128/mSphere.00209-20.8 TABLE S3 Significant edges (*q* < 0.05) between nodes (node 1 and 2) determined from the total 18S protist network. Nodes are described by ASV identification (ID) number, class, and species-level annotation. Additional information is shown for each association: edge type (copresence or exclusion), number of methods supporting the edge (2–5), method name (e.g., Spearman, Pearson, etc.), method scores, and merged *q* value. Download Table S3, XLSX file, 0.2 MB. Copyright © 2020 Anderson and Harvey. 2020 Anderson and Harvey This content is distributed under the terms of the Creative Commons Attribution 4.0 International license.

10.1128/mSphere.00209-20.9 TABLE S4 Significant edges (*q* < 0.05) determined from the filtered protist network, representing associations between protist groups (node 1) and Syndiniales (node 2). Nodes are described by ASV ID number, class, and species-level annotation. Information for each association is shown as in the total 18S protist network. We also show results of further annotation for Syndiniales ASVs using NCBI BLASTn. For each successful ASV annotation, query cover (%), E value, percent identity (%), and associated reference are shown. Download Table S4, XLSX file, 0.1 MB. Copyright © 2020 Anderson and Harvey. 2020 Anderson and Harvey This content is distributed under the terms of the Creative Commons Attribution 4.0 International license.

Positive (copresence) relationships, which may imply parasitism, were most common between Syndiniales and Dinophyceae ASVs, accounting for ∼70% of total edges between those groups (Fig. 3A and B). Syndiniales were also found to have positive relationships with Spirotrichea (e.g., *Tintinnidium* sp.) and other poorly represented taxa (<2 ASVs per class grouped into “other” category), such as those within the classes Trebouxiophyceae, Acantharea, and Filosa-Thecofilosea (Fig. 3; Table S4). Significant associations of Syndiniales with Syndiniales (15 total edges) as well as Syndiniales with abiotic factors were less frequent than associations with other protist groups (Fig. 3B).

### Syndiniales-Dinophyceae ASV relationships.

**FIG 4:**
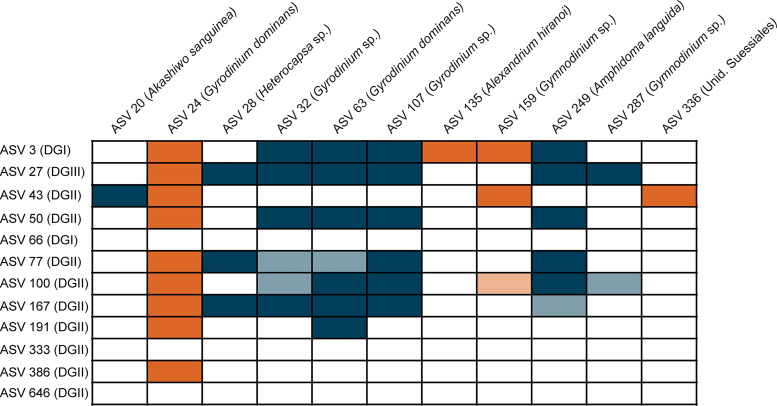
Diagram depicting the presence or absence of Syndiniales-Dinophyceae ASV pairings observed in the filtered network. Colored squares represent either a positive (copresence; blue) or negative (exclusion; orange) association for the respective pairing. Syndiniales parasites are referenced by group (Dino-Group [DG] I, II, or III), while species annotation is shown for dinoflagellates. Dinophyceae ASV 336 represents an unidentified Suessiales taxon. Dark shaded boxes represent edges with merged *q* values of <0.001, while lighter boxes reflect *q* values of <0.05.

**FIG 5:**
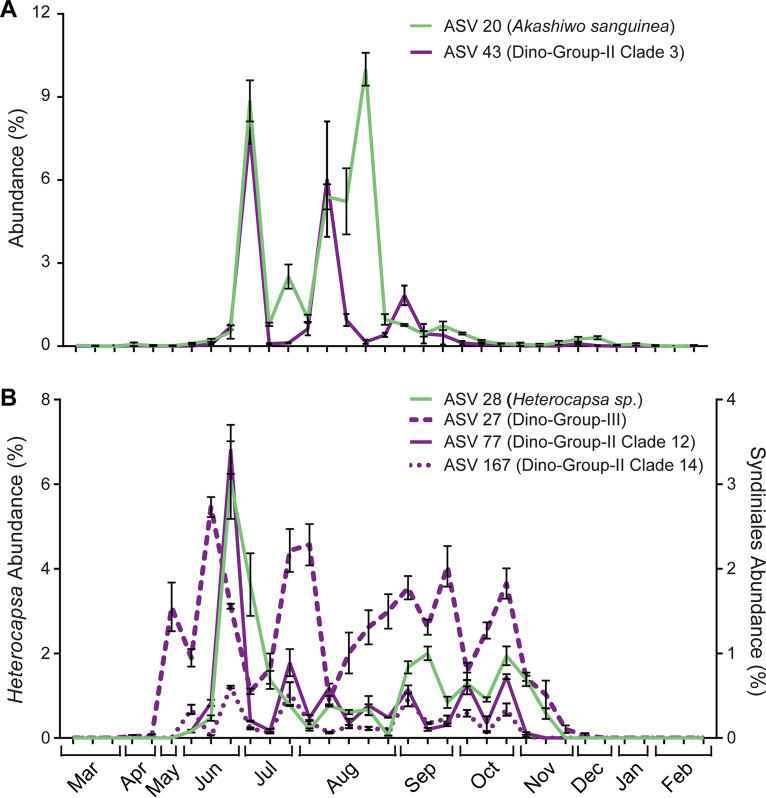
Relative abundance (%) plots of specific Syndiniales (purple) and Dinophyceae (green) ASV pairings that were found over the year in the estuary. Positive interactions rarely involved a one-to-one interaction (A), more often involving a single Syndiniales or Dinophyceae ASV interacting with >2 ASVs from the other group (B). All ASV-ASV interactions were significant (*q* values < 0.05) and derived from the filtered network. Error bars represent standard deviations from replicate sample means.

Given the large amount of positive Syndiniales-Dinophyceae associations, we constructed a presence/absence matrix of all significant edges found in the network between these groups to better examine potential parasite-host dynamics (Fig. 4). Positive and significant (*q* value < 0.05) associations were revealed between Syndiniales ASVs and ASVs from a range of dinoflagellates, including those from the genera *Gyrodinium*, *Gymnodinium*, *Heterocapsa*, *Akashiwo*, and *Amphidoma* (Fig. 4; see also Fig. S4). We observed a single example of a one-to-one positive relationship between Dinophyceae ASV 20 (Akashiwo sanguinea) and Syndiniales ASV 43 (Dino-Group II, clade 3), which interestingly, was further identified via BLASTn as an *Amoebophyra* strain infecting *A*. *sanguinea* (100% identity) (Table S4). Temporal dynamics of ASV 20 and 43 were strongly correlated over the year (Spearman *r *> 0.8; Pearson *r *> 0.6), with this combined ASV pairing accounting for up to ∼18% of protist relative abundance in June to October (Fig. 5A). Other Syndiniales ASVs included in the network were either identified to previously annotated *Amoebophyra* sp. sequences (via BLASTn) infecting Karlodinium micrum (90 to 91% identity; ASV 50 and 386) and Cochlodinium polykrikoides (98% to 99% identity; ASV 77 and 646) or were unassigned at the genus level (Table S4).

10.1128/mSphere.00209-20.4 FIG S4 Relative abundance (%) plots of specific Syndiniales (purple) and Dinophyceae (green) ASVs over the year in the estuary taken from the filtered network. All pairings (A to F) represent positive and significant (*q* values < 0.05) associations between groups. Error bars represent standard deviations from replicate sample means. Download FIG S4, TIF file, 1.5 MB. Copyright © 2020 Anderson and Harvey. 2020 Anderson and Harvey This content is distributed under the terms of the Creative Commons Attribution 4.0 International license.

Most often, a single Dinophyceae or Syndiniales ASV was positively associated with >2 ASVs from the other group (Fig. 4). For instance, positive relationships were observed between Dinophyceae ASV 28 (*Heterocapsa* sp.) and three different Syndiniales sequences, ASV 27 (Dino-Group III), ASV 77 (Dino-Group II, clade 12), and ASV 167 (Dino-Group II, clade 14), which resulted in a strong correlation (Spearman *r *> 0.7) between all ASV pairings over the year (Fig. 5B). Strong correlations (Spearman *r *> 0.6) between Syndiniales and ASVs in other major protists groups, such as Bacillariophyta, were also well represented. For example, Syndiniales ASV 27 (Dino-Group III) exhibited a strong negative relationship with Bacillariophyta ASV 210 (Thalassiosira minuscula) (see Fig. S5A), while a positive interaction was measured between Syndiniales ASV 3 (Dino-Group I, clade 1) and Bacillariophyta ASV 19 (*Cyclotella* sp.) (Fig. S5B).

10.1128/mSphere.00209-20.5 FIG S5 Relative abundance (%) plots of specific Syndiniales (purple) and Bacillariophyta (green) ASVs over the year in the estuary, representing a single exclusion (A) or copresence relationship (B). ASV associations were significant (*q* values < 0.05) and derived from the filtered network. Error bars represent standard deviations from replicate sample means. Download FIG S5, TIF file, 1.4 MB. Copyright © 2020 Anderson and Harvey. 2020 Anderson and Harvey This content is distributed under the terms of the Creative Commons Attribution 4.0 International license.

## DISCUSSION

Parasitism is an important source of mortality within marine protist communities (8), though it is seldom accounted for in ecosystem and biogeochemical models (2). Syndiniales are a diverse group of protist parasites that often dominate 18S rRNA relative abundance (11–13), infect a range of dinoflagellates and ciliates (20, 27), and can terminate or prevent coastal plankton blooms (31, 38). Studies of parasite-host interactions have typically involved few taxa—*Amoebophyra* spp. and dinoflagellates—revealing complex parasitic relationships with various degrees of infectivity and host specificity (18, 32). However, it has been difficult to characterize parasite-host infection dynamics in the marine environment, as most Syndiniales species remain unclassified and have yet to be established in culture (11). Additionally, poor resolution of Syndiniales communities over time has complicated our understanding of abiotic or biotic drivers of these parasite populations. We employed weekly 18S rRNA amplicon sequencing over a full year in the Skidaway River Estuary (Georgia), which allowed us to identify temporal shifts in Syndiniales abundance and composition relative to those of other major protist groups in the estuary. Furthermore, using relative abundance of dominant ASVs, we constructed a co-occurrence network to infer potential parasite-host interactions between Syndiniales and other protists. Our findings provide baseline information into temporal parasite dynamics, which will be important for accurate assessment of their ecological roles within marine systems and in ecosystem models.

### Temporal shifts in Syndiniales.

While prior work has noted seasonal shifts in Syndiniales relative abundance and infection prevalence (percentage of infected host) via monthly sampling (16, 22), here we report strong temporal dynamics of Syndiniales at a higher sampling resolution (∼weekly) in a subtropical estuary, with parasites accounting for 7% to 28% of total protist relative abundance from June to October compared to <6% for the remainder of the year. Relative abundances of other major class level protists (e.g., Bacillariophyta and Dinophyceae) were more stable over the year, though temporal trends in total protist composition and diversity mirrored that of Syndiniales only, with communities forming a distinct cluster with elevated surface temperatures (23 to 31°C). Despite temporal shifts in composition and environmental variables, Shannon diversity remained relatively stable over the year, which has been noted elsewhere (39, 40) and may indicate thresholds for species richness within protist communities. We acknowledge that comparing relative abundance among eukaryotic taxa remains tenuous, particularly as copy numbers per cell of the 18S rRNA gene can vary 4-fold among protist groups (41). While copy numbers roughly scale to biovolume (42, 43), this relationship may not hold for certain protist groups such as alveolates (including Syndiniales), leading to overestimations of relative abundance (41, 44). For Syndiniales, this is further complicated by its life cycle, as trophonts are expected to have higher gene copy numbers than smaller dinospores. While higher gene copy numbers may have influenced the relative abundance of Syndiniales in our study, free-living dinospores can reach concentrations of 800 to 1,500 cells ml^−1^ in estuaries (25, 31), and so the elevated Syndiniales relative abundance that we observed in summer to fall may have accurately reflected absolute increases in spore abundance or biomass. Nevertheless, we observed clear temporal variability in Syndiniales and, in general, present data on temporal shifts within a class to mitigate biases from gene copy number (17, 45).

Based on CCA and PLS analyses, temperature and nutrients (especially silicate) were identified as important abiotic correlates with Syndiniales relative abundance and composition. Temperature has been shown to determine physiological rates of marine microbial eukaryotes (4, 46), and warmer temperatures may accelerate the infectivity of Syndiniales and dinospore production (20, 26). Temperature has also been shown to influence parasites of diatoms, with infection persisting only at temperatures of >4°C (47). Conversely, high relative abundances of Syndiniales sequences have been observed in polar regions at temperatures of <1°C (13, 14). This may reflect the presence of strain- or species-specific differences in thermal tolerance or distribution patterns among Syndiniales; however, the role of water temperature in mediating Syndiniales-host interactions remains unclear. Environmental factors we included poorly explained the variance in parasite composition (19%), which suggests abiotic factors may play a reduced role in structuring parasite communities or that other factors not considered here (e.g., turbulence or mixing) may be important (26). We also observed strong collinearity of temperature and nutrients in the estuary, making it difficult to ascribe shifts in protist communities to any single environmental factor. It is more likely that temporal dynamics of Syndiniales in the estuary were driven by biological interactions between taxa instead of abiotic variables, which has been observed in other microbial networks (48, 49) and in our network, where significant abiotic interactions were scarce. Thus, while effects of abiotic factors on Syndiniales warrant further investigation (e.g., temperature effects), determining Syndiniales infection dynamics in the natural environment may rely more on the interactions that occur between parasites and hosts.

Syndiniales are highly influenced by host biomass and density (20, 24, 50), with parasitic encounter and infection expected to decrease under conditions that are unfavorable for autotrophic, heterotrophic, or mixotrophic hosts (e.g., reduced temperature and nutrients or limited prey availability). For instance, *Amoebophyra* spp. (Syndiniales Dino-Group II) have been shown to produce 3 to 4 times fewer dinospores per infected host cell and have lower infectivity under nutrient-deplete conditions than under nutrient-replete conditions (19). The reduced Syndiniales relative abundance we observed in winter and spring may have been driven by poor environmental conditions for host cells (e.g., low temperature and nutrients), inhibiting accumulation of host biomass available for parasitism. Prior work in the Skidaway River Estuary (37) measured significantly lower phytoplankton accumulation rates in winter to spring compared to that in summer to fall (−0.16 to 0.28 day^−1^ versus 0.48 to 1.09 day^−1^, respectively), with such shifts in biomass accumulation potentially affecting host/parasite ratios and encounter rates. Despite low relative abundance of parasites in winter to spring (0.01% to 6%), Syndiniales may have survived during this time by residing within host cysts or switching hosts (18, 51), allowing them to exploit intermittent or seasonal blooms. Though we cannot verify such survival strategies in our analyses, the Skidaway River Estuary is a shallow site (4 to 6 m) with strong benthic-pelagic coupling (34, 52), which would facilitate the vertical transport of dinoflagellate cysts (and enclosed parasites) to the sediment. Additional temporal examinations of Syndiniales are needed to establish drivers of natural parasite communities and should take into consideration relative and absolute shifts in parasite-host systems, as well as parasite survival strategies under unfavorable conditions.

### Syndiniales interactions via network analysis.

Network inference via co-occurrence analysis has become a widely applied technique in microbial ecology (35, 36) and has been used to predict significant relationships or interactions among protist sequences (53–55). Importantly, positive interactions inferred via co-occurrence networks can represent ecologically meaningful associations between ASVs (e.g., parasitism, predation, or mutualism) or simply an overlapping niche between taxa (35). We focused on positive correlations between Syndiniales and protists that may indicate parasitism (15). While such networks can represent powerful tools for generating hypotheses, we acknowledge the limited causality of such interactions and the need to confirm them via previous literature, interaction databases (e.g., Protist Interaction DAtabase [9]), or empirical methods such as lab culturing, microscopy, or other omics-based applications (44).

As with other temporal protist networks (15–17, 53), Syndiniales were well represented in our yearly network, accounting for 20% of all significant interactions between protist taxa. Positive interactions were most common between Syndiniales and Dinophyceae ASVs (e.g., *Gyrodinium*, *Gymnodinium*, *Heterocapsa*, and *Akashiwo*), which confirms the high frequency of Syndiniales-Dinophyceae interactions in previous networks (15) and direct observations of parasitic infection within these same dinoflagellate species in prior lab and field work (20, 23). Though currently unsupported in the literature, we found multiple Syndiniales ASVs to positively interact with a toxin-producing (azaspiracid) dinoflagellate species, Amphidoma languida (56). This may represent a potential parasitic relationship, as epidemic outbreaks of *Amoebophyra* spp. have been reported following blooms of other toxic dinoflagellates (e.g., *Alexandrium* and *Dinophysis*) (20, 57), and in general, parasites may exhibit tolerance toward toxin production (58). Further insight into the suspected interaction and coevolution of Syndiniales parasites and toxin-producing dinoflagellates is essential given the potential top-down control of harmful blooms via parasitism and related effects on carbon cycling within marine ecosystems (31, 58). While no direct evidence of infection was verified in this study, our findings of abundant Syndiniales-Dinophyceae interactions in the network, including those with harmful plankton, reinforces the importance of these parasitic interactions in coastal protist communities (20).

We also observed positive interactions between Syndiniales and known host taxa other than dinoflagellates, including Spirotrichea (e.g., *Tintinnidium* sp. and unidentified Strombidiidae) and Acantharea (unidentified radiolarians), both of which have been associated with Syndiniales in phylogenetic and empirical work (27, 59). Tintinnid ciliates are commonly recognized hosts of the Syndiniales genera, *Euduboscquella*, with infection being prevalent in coastal ciliate populations (27, 28, 60). Though *Euduboscquella* spp. were not identified among abundant Syndiniales sequences in our network, the presence of positive relationships between Syndiniales and ciliates may suggest parasitic relationships between these groups in the estuary. We recognize that positive associations with ciliates could also imply predator-prey relationships, as there is evidence of ciliates consuming Syndiniales dinospores (61, 62). Likewise, flagellates and dinoflagellates may also contribute to consumption of dinospores, given the overlap in size range of spores with certain phytoplankton prey (63). Understanding ecological interactions between Syndiniales and dinoflagellates, as well as those formed between parasites and other protist groups, will enhance our understanding of parasite host range and top-down impacts of parasitism.

The most common interactions were found between Syndiniales and Bacillariophyta, though such associations were largely negative (75%), indicative of exclusion. A recent network study, which summarized spatial interactions from the Tara Oceans interactome (15), also found a high proportion of negative associations between Syndiniales and diatoms (64). Diatoms may prevent co-occurrence with harmful protists (e.g., grazers or parasites), reflective of trait-based mechanisms (e.g., silicate cell walls, chain formation, or toxic oxylipins) adapted to avoid mortality (64, 65). We cannot rule out that negative interactions between Syndiniales and diatoms may indicate a type of resource competition (either direct or indirect) between taxa, as nutrients such as silicate were important in explaining patterns in Syndiniales composition. It is also possible that Syndiniales and diatoms occupied different ecological niches within the estuary. Positive interactions that were detected between these groups remain ambiguous, as there has yet to be empirical evidence of Syndiniales infecting diatoms. However, Syndiniales have been shown to positively associate with diatoms in other co-occurrence networks or single-cell studies (15, 16, 66), and diatoms are known hosts of other parasitic protists and fungi (8, 47), which warrants further investigation into their infection dynamics.

Our findings on temporal dynamics of Syndiniales represents an important baseline to form data-driven hypotheses on specific parasite-host interactions, seasonality effects, and potential abiotic drivers of parasites, all of which can be explored in more detail using a range of lab and field techniques. For example, microscopy can be used to visualize all stages of Syndiniales infection (dinospores to trophont), as parasites emit green autofluorescence under blue light excitation (26, 51). These techniques may extend to FlowCam imaging, which has an option to trigger cell autofluorescence and is more rapid than traditional microscopy (67). Quantitatively, there may be limitations to this approach that warrant further testing, as the FlowCam may not enumerate <10-μm dinospores. Isolation and sequencing of single amplified genomes (SAGs) via cell sorting (68) may also be used to identify dinoflagellates and parasite symbionts (44), though such applications remain poorly tested for parasite-host interactions. Additional omics tools, such as transcriptomics, may be used to inform *in situ* parasite-host interactions, distinguishing between different stages of Syndiniales (e.g., intracellular trophont versus free-living dinospores) based on patterns in gene expression and testing how core genes involved in parasitic infection (e.g., host recognition or attachment) may be differentially expressed under certain abiotic or biotic conditions (69). Indeed, 18S profiling of a larger size fraction (e.g., <200 μm) cannot distinguish between Syndiniales sequences derived from trophont or dinospore stages, and so fractionation of 18S samples (e.g., <5 μm) may be useful to examine the relative abundance of Syndiniales across multiple size fractions (66). In all, these alternative techniques will be important to complement future 18S amplicon studies and verify temporal patterns and parasite-host interactions proposed via co-occurrence networks.

### Syndiniales host specificity.

Given the high frequency of positive Syndiniales-Dinophyceae associations and the often-observed infection of coastal dinoflagellates (20, 28), we chose to further examine host specificity of these associations. We found only one example of a highly specific positive interaction involving Dinophyceae ASV 20 (*Akashiwo sanguinea*) and Syndiniales ASV 43 (identified as *Amoebophyra* sp. infecting *A*. *sanguinea*), confirming previous accounts of this highly specific parasitic relationship (18, 23). *A*. *sanguinea* is common in the Skidaway River (70), and populations of this harmful species can exceed 1,000 μg C liter^−1^ in summer (71), during which time *A*. *sanguinea* is likely susceptible to parasitic infection. It was more common for Syndiniales ASVs in our network to be associated with multiple dinoflagellates or for multiple Syndiniales to interact with the same dinoflagellate, suggesting flexible parasite-host dynamics. Other studies have noted a generalist infection strategy among Syndiniales and putative dinoflagellate hosts (11, 16, 57), which may explain their ubiquity in marine systems, including both open ocean and coastal regions, as well as their quick response to elevated host density. Though we focused on only the most abundant Syndiniales and protist sequences for our yearly network, we recognize there may be ephemeral parasite-host interactions that were overlooked (e.g., in winter to spring) and may reflect more specific interactions (31). There are likely a myriad of infection strategies displayed among Syndiniales taxa that enables parasites to exploit a range of plankton host conditions in both coastal and open oceans.

Despite the diversity and ubiquity of Syndiniales in the global oceans (11, 12), large gaps in knowledge of their temporal patterns and infection dynamics has made it difficult to place parasitism within food web or ecosystem models (72). In the data presented here, Syndiniales were temporally variable over a full year and well represented in our protist network, exhibiting positive interactions with a range of previously known hosts (e.g., dinoflagellates and ciliates), which largely involved >2 taxa associated with each parasite ASV. Interactions between Syndiniales and diatoms were most abundant, largely representing negative associations that may imply possible competition or avoidance mechanisms between taxa (64). Though temperature emerged as an important factor predicting Syndiniales relative abundance, abiotic factors were limited in the network compared to biotic interactions. Given their importance within the protist community and broad host range, parasitism by Syndiniales is expected to have significant consequences for carbon cycling and ecosystem functioning in marine systems (8, 11). In the Skidaway River Estuary, recent work has identified microzooplankton grazing as the primary source of primary production loss (37), though grazing rates were seasonal and at times did not control plankton biomass (positive accumulation in summer), indicating that other forms of mortality that were unaccounted for in the dilution experiments, such as parasitism, may have contributed to phytoplankton loss. Determining the contribution of parasitism to plankton mortality will be important, especially in relation to other forms of mortality (e.g., predation or viral lysis), which have contrasting effects on carbon and nutrient cycling in marine food webs. Gathering baseline information on the infection dynamics of Syndiniales is therefore critical to accurately predict the impact of parasitism on plankton populations and biogeochemical cycling.

## MATERIALS AND METHODS

### Sample collection.

Surface water (1 m) was collected approximately every 1 to 2 weeks over one year (16 March 2017 to 21 February 2018) from the Skidaway River Estuary (latitude, 31°59′25.7″N; longitude, 81°01′19.7″W), which is accessible via the Skidaway Institute of Oceanography (GA, USA). Sampling always occurred at high tide. Seawater (10 liters) was collected via Niskin bottles, filtered through a 200-μm mesh (to limit mesozooplankton), and immediately transferred to the laboratory in clean 20-liter carboys for filtration. Due to high particulate content in the estuary (see reference 52) and to avoid filter clogging, smaller volumes (250 to 1,000 ml) were filtered onto triplicate 47-mm 0.2-μm polycarbonate filters (Millipore) and stored at −80°C until DNA extraction. Environmental factors, including temperature, salinity, dissolved oxygen, chlorophyll, particulate organic carbon and nitrogen, and nutrients were measured in a previous temporal study of plankton mortality rates in the estuary (37) and included here to explore correlations with protists (raw data available in Table S1 in the supplemental material). Nutrients and organic carbon were not measured on 6 September 2017, and so this day was removed prior to correlation analyses.

### DNA extraction and sequencing preparation.

Triplicate filters were thawed on ice, and total DNA was extracted with the Qiagen DNeasy PowerSoil kit (Qiagen) as per the manufacturer’s instructions, which included repeated steps of bead beating for mechanical lysis of cells and a final elution step in 10 mM Tris-HCl (pH 8.5). The concentration of eluted DNA was tested for each sample using a Qubit dsDNA HS kit (Thermo Scientific), with yields ranging from 2 to 5 ng μl^−1^. The V4 region of the 18S rRNA gene was targeted using primers from Stoeck et al. (73): forward (5′-CCAGCASCYGCGGTAATTCC-3′) and reverse (5′-ACTTTCGTTCTTGATYRA-3′). We chose the V4 region because its length (ca. 400 bp) has been shown to increase phylogenetic resolution and diversity estimates compared to those with shorter regions of the 18S rRNA gene (74); however, primer sets that target other 18S regions (V9 or V4-V5) may better represent certain taxonomic groups such as haptophytes (75). Amplicon libraries were prepared using a two-step PCR approach with the following thermocycling parameters: initial denaturation at 98°C for 2 min, 10 cycles of 98°C for 10 s, 53°C for 30 s, and 72°C for 30 s, followed by 15 cycles of 98°C for 10 s, 48°C for 30 s, and 72°C for 30 s, and a final extension of 72°C for 2 min (53, 76). PCR products from the initial run were purified using AMPure XP Beads (Beckman Coulter, A63881), and a second PCR step was performed using duel Illumina indices (P5 and P7). Amplicon libraries were sequenced using an Illumina Miseq (2 × 250 bp) at the Georgia Genomics and Bioinformatics Core at the University of Georgia.

### Analysis of 18S sequences.

Amplicon sequence variants (ASVs) were inferred from raw sequences using the DADA2 program (https://benjjneb.github.io/dada2/index.html, V1.12) in R (https://cran.r-project.org, V3.6.1), which allows for high-resolution (1- to 2-bp difference) identification of sequences (77). After inspection of read quality profiles, the following parameters were used to filter sequences (e.g., remove primers and phiX contamination) while maintaining sufficient overlap between paired reads: trimLeft = c(18, 20), maxN = 0, maxEE = c(2), truncQ = 2, rm.phix = TRUE. Parametric error models were applied to the first 100 million bases of the forward and reverse reads, and following dereplication, error rates were used to infer ASVs. Finally, paired reads were merged and reads with unexpected lengths (<1% of reads) and chimeras (ca. 2% of reads) were removed using default DADA2 parameters.

Merged 18S rRNA sequences were classified against the Protistan Ribosomal Reference (PR2; V4.11.1 [78]) database using the “assignTaxonomy” function in DADA2, which implements a naive Bayesian classifier method (79). Often, species level annotation was missing from the ASV table (e.g., *Chaetoceros* sp.), and so we added this information if the genus level was annotated properly (e.g., *Chaetoceros*). Additionally, we searched the most abundant Syndiniales sequences (distinguished from the network and present in >50% of all samples) in the NCBI database with BLASTn to assess further classification to genus level (e.g., *Amoebophyra*). Over the 33 sampling days included in the data set, we had an average of 62,529 sequence reads per replicated sample (range = 29,160 to 104,327), which corresponded to a total of 9,768 ASVs over the year (548 on average per replicate).

Tables produced by the DADA2 pipeline were imported into the phyloseq package in R (V1.28.0 [80]) to evaluate diversity metrics and community composition. Prior to diversity analysis, 18S ASVs that could not be identified at the supergroup level were removed. As we focused on diversity and associations among protists, ASVs were filtered to exclude material derived from metazoans and expected parasites of metazoans that resided within Syndiniales group IV (11). After these filtering steps, a total of 8,698 ASVs remained across all samples. Using the filtered ASV table, rarefaction curves were generated for each replicate in each month using the ggrare function (see Fig. S1). Diversity plots were made for all ASVs (i.e., total 18S community) and for only Syndiniales ASVs. Tukey box plots displaying Shannon alpha diversity were produced using the plot_richness function. For remaining analysis, global singletons were removed (590 ASVs), and samples were normalized by calculating relative abundance for each ASV on a given day. After normalizing abundance, nonmetric dimensional scaling (NMDS) ordination was performed using Bray-Curtis distances. Temporal shifts in the community at the class and order levels (e.g., within Syndiniales) were visualized using the plot_bar function in phyloseq and included groups with relative abundance >5% on any given day. The filtered ASV table containing reference sequences, taxonomic identification, and relative abundances for each replicate sample are provided in Table S2.

The correlation between environmental factors and composition (total 18S and within Syndiniales) was explored using canonical correspondence analysis (CCA) using the vegan package in R (V2.5 [81]). Environmental factors were log-transformed and the ordistep function in vegan was used to test the significance of factors to the ordination in a stepwise manner. Contributions of environmental factors were added as arrows to the ordination plot. To further assess the importance of environmental factors in predicting class-level relative abundances, a partial least-squares (PLS) regression was applied using the R package pls (V2.7 [82]). PLS was included to mitigate collinearity of environmental factors, as a strong correlation between temperature and silicate was observed in the estuary (Spearman *r *> 0.8 [37]). Separate models were run using the predictor variables (environmental factors), and class-level relative abundances were averaged for each major protist group. All variables were log transformed and standardized by centering and scaling to unit variance (83). After initial validation, a 3-component model was used for each protist group, capturing 57% to 65% of the variance in the predictor variables. Variable influence on the projection (VIP) scores were calculated for each predictor variable, with a VIP of >1 considered more important to the model (83).

### Protist network analysis.

Co-occurrence networks were constructed using CoNet (V1.1.1 beta), which enabled visualization of significant interactions between Syndiniales and protist ASVs (84). CoNet is an ensemble-based approach that combines multiple pairwise measures to restrict prediction of false-positive relationships. Each measure assigns a positive (copresence) or negative (exclusion) sign to a predicted relationship, which reflects whether relative abundance distributions of any two ASVs are significantly more similar or dissimilar than expected at random (84). To focus on the most prevalent taxa and avoid ambiguous relationships, only ASVs present in >50% of all samples (152 ASVs) were included in the network. Filtered ASV tables were normalized to relative abundance, and relationships between taxa and environmental variables were explored by loading metadata as a separate matrix. Sampling days had uneven intervals between them, and so we did not consider time-lagged correlations.

We followed general CoNet settings as described in Faust and Raes (85). Briefly, pairwise relationships were explored between ASVs using five measures (Bray Curtis and Kullback-Leibler dissimilarities, Pearson and Spearman correlations, and mutual information similarity), with each measure contributing 1,000 positive and negative edges. To alleviate compositional bias of sequencing data, a renormalization ReBoot routine was applied (84), which generated 100 permutation and bootstrap scores for each edge and measure. Measure-specific *P* values were then computed for each edge as the probability of observing the null value (i.e., mean of edge score distribution) under a Gauss curve generated from the bootstrap distribution (85). After merging of *P* values (resulting in *q* values) using Brown’s method (86) and correcting for multiple testing with Benjamini-Hochberg’s procedure, edges with *q* values of <0.05 were retained. Edges were only retained if they were supported by two or more measures or were unanimously given the same sign by all measures. Circular networks were initially visualized in Cytoscape (V3.7.1 [87]), and then network information (edges and nodes) was exported and visualized as circos plots in the R package, circlize (88). Nodes (represented by ASVs) were grouped and colored based on class-level annotation. The final network was filtered to include only positive and negative edges measured between Syndiniales ASVs and other major protist groups or abiotic variables. We focused on positive interactions between Syndiniales and protists that may infer parasitism, though given the limited causality of correlation networks (44), we relied on prior literature to confirm parasitic interactions indicated by the network.

### Data availability.

Demultiplexed raw sequences were deposited in NCBI SRA under BioProject ID PRJNA575563 (Biosample accession numbers SAMN12901176 to SAMN12901144). Additionally, R code used to infer ASVs and generate figures and downstream analyses have been made available on GitHub (https://github.com/sra34/SkIOprotists). The GitHub repository also includes ancillary files for metadata, co-occurrence networks, and ASV count and taxonomy tables.
